# Terahertz Spin‐to‐Charge Conversion by Interfacial Skew Scattering in Metallic Bilayers

**DOI:** 10.1002/adma.202006281

**Published:** 2021-01-27

**Authors:** Oliver Gueckstock, Lukáš Nádvorník, Martin Gradhand, Tom Sebastian Seifert, Genaro Bierhance, Reza Rouzegar, Martin Wolf, Mehran Vafaee, Joel Cramer, Maria Andromachi Syskaki, Georg Woltersdorf, Ingrid Mertig, Gerhard Jakob, Mathias Kläui, Tobias Kampfrath

**Affiliations:** ^1^ Department of Physics Freie Universität Berlin Arnimallee 14 14195 Berlin Germany; ^2^ Department of Physical Chemistry Fritz Haber Institute of the Max Planck Society Faradayweg 4–6 14195 Berlin Germany; ^3^ Faculty of Mathematics and Physics Charles University Ke Karlovu 2027/3 Prague 12116 Czech Republic; ^4^ School of Physics University of Bristol Tyndall Avenue Bristol BS8 1TL UK; ^5^ Institut für Physik Johannes‐Gutenberg‐Universität Mainz Staudingerweg 7 55128 Mainz Germany; ^6^ Department of Materials ETH Zürich Hönggerbergring 64 Zürich 8093 Switzerland; ^7^ Singulus Technologies AG 63796 Kahl am Main Germany; ^8^ Institut für Physik Martin‐Luther‐Universität Halle Von‐Danckelmann‐Platz 06120 Halle Germany

**Keywords:** interface, skew scattering, spin‐to‐charge conversion, terahertz emission spectroscopy

## Abstract

The efficient conversion of spin to charge transport and vice versa is of major relevance for the detection and generation of spin currents in spin‐based electronics. Interfaces of heterostructures are known to have a marked impact on this process. Here, terahertz (THz) emission spectroscopy is used to study ultrafast spin‐to‐charge‐current conversion (S2C) in about 50 prototypical F|N bilayers consisting of a ferromagnetic layer F (e.g., Ni_81_Fe_19_, Co, or Fe) and a nonmagnetic layer N with strong (Pt) or weak (Cu and Al) spin‐orbit coupling. Varying the structure of the F/N interface leads to a drastic change in the amplitude and even inversion of the polarity of the THz charge current. Remarkably, when N is a material with small spin Hall angle, a dominant interface contribution to the ultrafast charge current is found. Its magnitude amounts to as much as about 20% of that found in the F|Pt reference sample. Symmetry arguments and first‐principles calculations strongly suggest that the interfacial S2C arises from skew scattering of spin‐polarized electrons at interface imperfections. The results highlight the potential of skew scattering for interfacial S2C and propose a promising route to enhanced S2C by tailored interfaces at all frequencies from DC to terahertz.

## Graphical abstract

Terahertz emission spectroscopy is used to study spin‐to‐charge conversion (S2C) in ferromagnetic (F)|nonmagnetic (N) bilayer metal thin films. The measurements indicate that S2C at the F/N interfaces can become comparable to the overall S2C and that the sign is drastically affected by interface modifications. A possible extrinsic S2C mechanism, skew scattering of spin‐polarized electrons at interfacial imperfections, is suggested.

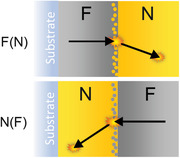

The spin of the electron bears large potential as information carrier in future electronics.^[^ ^1^ ^]^ An essential operation in spintronic devices is the transformation of spin into charge currents and vice versa.^[^ ^2^ ^]^ A generic structure for studying such spin‐to‐charge‐current conversion (S2C) is the prototypical bilayer of **Figure** **1**a: A spin current with electron‐number density *j*_s_ flowing along the *z* direction is converted into a transverse charge current with density *j*_c_. S2C and its inverse process facilitate the efficient detection and generation of spin currents, the central element of spintronic operations.^[^ ^2^ ^]^ A highly relevant application of the resulting spin current is to exert torque on nearby spins to switch their magnetic order,^[^ ^3^ ^]^ even with terahertz (THz) fields.^[^ ^4^ ^]^

**Figure 1:**
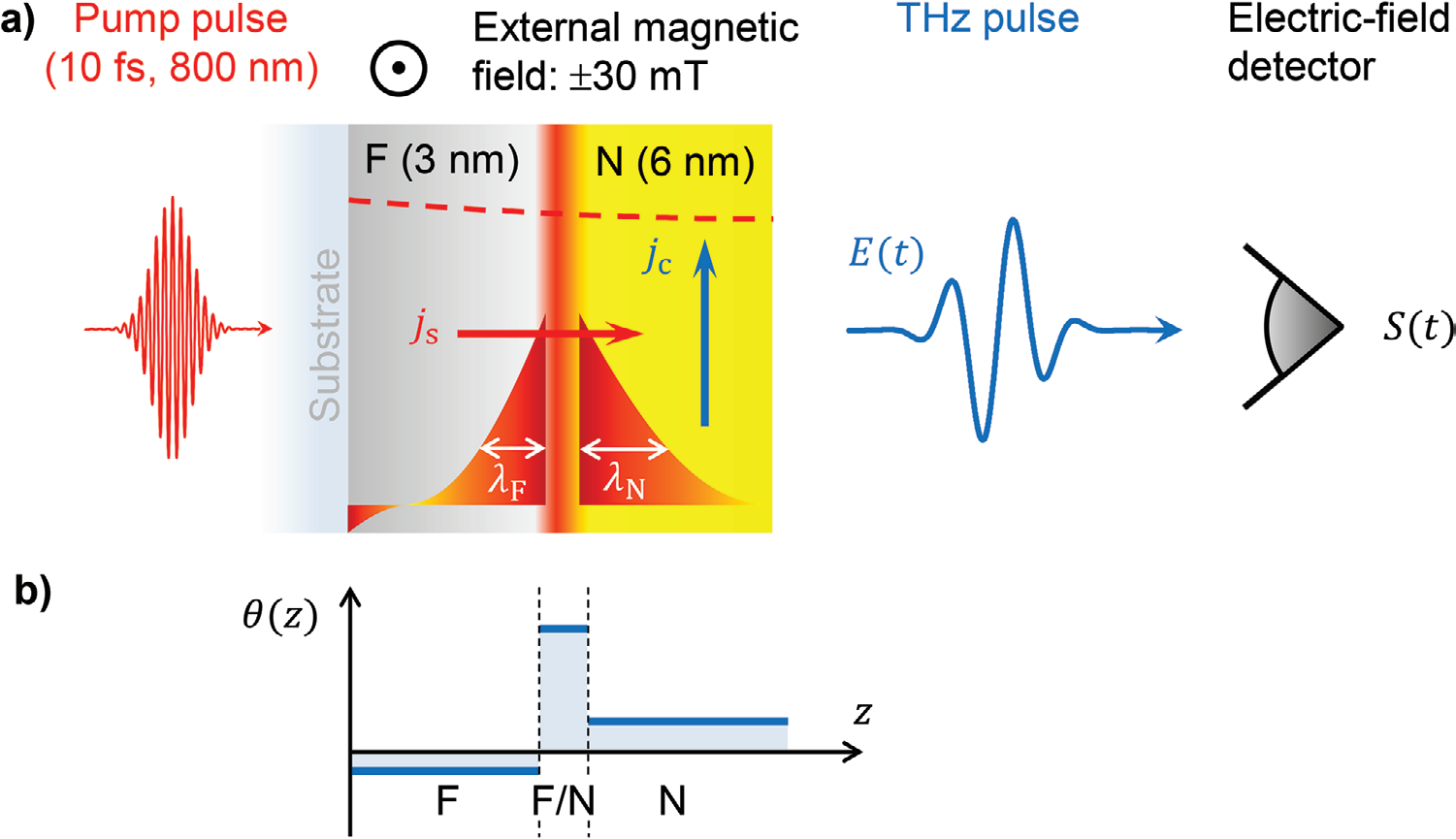
Photoinduced spin transport and spin‐to‐charge current conversion (S2C) in F|N bilayers. a) Side view of a F|N bilayer consisting of a ferromagnetic metal layer (F) and an adjacent nonmagnetic metal layer (N). A femtosecond laser pulse excites the metal stack from the substrate side. The calculated pump‐field profile inside the bilayer is indicated by the red dashed line. The optical excitation drives a spin current from F to N whose density *j*_s_(*z*,*t*) decays on the length scales λ_F_ and λ_N_ as the distance from the F/N interface increases. Spin current also flows in the vicinity of the F/substrate interface. At any position *z*, *j*_s_ is converted into a charge current with density *j*_c_, leading to the emission of a THz electromagnetic pulse. b) Example of a possible *z*‐dependence of the local S2C strength θ(*z*) in the F|N bilayer (Equation (1)). F/N denotes the interface region.

In a local picture, S2C may be described by the relationship

(1) $$\begin{array}{c} j_{c}\left(z\right)=\theta \left(z\right)j_{s}\left(z\right) \end{array}$$

where the spin Hall angle (SHA) θ(*z*) quantifies the strength of S2C at position *z*. Note that a nonvanishing θ(*z*) can occur at any plane *z* in a metal bilayer (Figure 1b): In the bulk of the ferromagnet (F), the bulk of the nonmagnet (N), and at the two interfaces of the F layer. Major S2C effects are the inverse spin Hall effect (ISHE)^[^ ^2^ ^]^ in nonmagnetic materials and ferro‐ or ferrimagnets and the inverse Rashba–Edelstein effect (IREE),^[^ ^5^, ^6^ ^]^ the latter only occurring in regions with broken inversion symmetry like interfaces.

Recently, the operational speed of S2C was extended to ultrafast time scales using bilayer structures as that of Figure 1a. First, a femtosecond laser pulse was used to generate spin currents perpendicular to the plane through the ultrafast spin Seebeck effect^[^ ^7^ ^]^ and ultrafast superdiffusive spin currents.^[^ ^8^, ^9^, ^10^, ^11^, ^12^, ^13^, ^14^, ^15^, ^16^, ^17^, ^18^ ^]^ By means of S2C, the spin current was converted into an in‐plane ultrashort charge current burst giving rise to the emission of THz electromagnetic waves. This scheme has enabled new applications such as spintronic emitters of ultrashort THz electromagnetic pulses.^[^ ^8^, ^9^, ^10^, ^11^, ^12^, ^13^, ^14^, ^15^ ^]^

In view of these applied aspects, a fundamental understanding and, eventually, control of S2C are highly desirable. Extensive research indicates that the most efficient materials for bulk S2C conversion are still Pt and W,^[^ ^2^ ^]^ which mainly rely on the ISHE due to their strong spin–orbit coupling. To boost S2C, researchers have, therefore, started studying the role of interfaces. Recent works have shown that tailored interfaces of nonmagnetic materials such as the interface between Bi and Ag exhibit sizeable S2C due to the IREE at sub‐GHz frequencies^[^ ^19^ ^]^ and in the THz regime.^[^ ^5^, ^6^, ^20^, ^21^ ^]^ Recently, THz emission even from single ferromagnetic layers was observed and ascribed to interfacial effects.^[^ ^22^ ^]^ It is, thus, highly interesting to further explore interfacial S2C in terms of signatures beyond the IREE.

In this work, we study ultrafast laser‐driven S2C in the F|N bilayer model system. To identify possible contributions of the F/N interface, we: i) consider all combinations out of six F and three N materials with bulk S2C of different strength and sign and ii) modify the interface while leaving F and N bulk as unaffected as possible. In bilayers with N = Cu and Al, a surprisingly strong S2C is found, even though Cu and Al are known to have a negligible bulk ISHE. We show that S2C in these samples is drastically affected by modification of the interface. For example, in Ni_81_Fe_19_|Cu, the interface contribution is dominant and estimated to be as large as 20% of S2C in Ni_81_Fe_19_|Pt. Based on symmetry arguments and first‐principles calculations, we consistently assign the interfacial S2C observed here to skew scattering of spin‐polarized electrons at interface imperfections. Our results highlight a promising route to enhancing S2C by exploiting interface‐related conversion mechanisms.

*Experiment Design*: A number of methods to measure the strength of S2C of a given F|N bilayer sample are available.^[^ ^23^ ^]^ Here, we make use of THz emission spectroscopy for the following reasons: First, it features a large sample throughput per time, which is essential for the large number (≈50) of samples of our study. Second, THz emission spectroscopy can be applied to as‐grown bilayers without micro‐structuring. Finally, the high signal‐to‐noise ratio permits the investigation of samples with relatively small S2C strength.^[^ ^10^ ^]^ We emphasize that THz emission spectroscopy was shown to deliver values of the relative S2C conversion strength which are fully consistent with values extracted from established electrical techniques based on broadband ferromagnetic resonance,^[^ ^23^ ^]^ harmonic Hall measurements^[^ ^23^ ^]^ and the DC spin Seebeck effect.^[^ ^24^ ^]^

Our THz emission spectrometer is schematically shown in Figure 1a. A femtosecond laser pulse (energy 1 nJ, duration 10 fs, center wavelength 800 nm, repetition rate 80 MHz) excites an F|N bilayer from the substrate side and triggers a spin current with density *j*_s_(*z*,*t*) from F to N (Figure 1a). The longitudinal *j*_s_ is converted into a transverse charge current with density *j*_c_, thereby emitting electromagnetic radiation whose spectrum extends into the THz range. The transient electric field *E*(*t*) of the THz pulse is detected by electro‐optic sampling in the far‐zone, resulting in an electro‐optic signal waveform *S*(*t*) that is related to *E*(*t*) through a linear transfer function.^[^ ^7^, ^25^ ^]^

Our samples are metallic F|N bilayers with an MgO protective coating. They are deposited on glass substrates by sputtering, resulting in the sample structure glass (500 µm)||F(3 nm)|N(6 nm)|MgO(3 nm). To identify possible interface contributions to S2C, we first consider all combinations of six common ferromagnetic materials (such as Fe, Permalloy Py (Ni_81_Fe_19_), and Co) and three common nonmagnetic materials (Pt, Cu, and Al) with different magnitude and sign of their bulk S2C. In this way, we vary the S2C strength θ (Equation (1)) of the N layer from strong (Pt) to very weak (Al or Cu) and of the F layer from positive (Py, like Pt) to negative (Fe or Co).^[^ ^26^, ^27^, ^28^ ^]^ The direction of the F‐layer magnetization ± **M** is set by an external magnetic field that is sufficiently strong to saturate the sample magnetization. In a second step, we modify the F/N interface while leaving F and N bulk as unaffected as possible.

To further characterize our F|N bilayers, we measure their optical absorptance *A* and THz impedance *Z*. Both *A* and *Z* are important to normalize the measured THz emission signals, thereby enabling a direct comparison of the S2C strength between different samples.^[^ ^29^ ^]^

*Raw Data*: The THz waveforms seen in **Figure** **2**, which displays typical THz emission signal waveforms, were obtained from Py|N bilayers for N being Pt, Cu, and Al. We focus on the signal component odd in the sample magnetization,

(2) $$\begin{array}{c} S\left(t\right)=S\left(t,M\right)-S\left(t,-M\right) \end{array}$$

which strongly suppresses all non‐magnetic contributions to the signal. It is typically at least one order of magnitude larger than the even signal *S*(*t*, **M**) + *S*(*t*, −**M**) (see Figure S1a in the Supporting Information). The signal strengths observed for Py|Cu and Py|Al are quite sizeable relative to that of Py|Pt, which is known to exhibit strong S2C.

**Figure 2:**
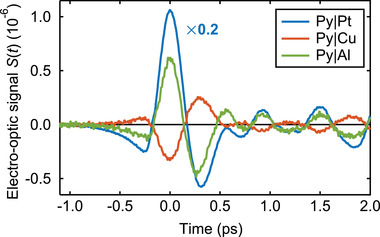
THz emission from Py|N. The curves show time‐domain electro‐optic signals *S*(*t*) of THz pulses emitted from photoexcited Py|N bilayers, where N is Pt, Cu, or Al. All shown signals are odd with respect to the sample magnetization **M** (see Equation (2)). Note the rescaling factor for Pt.

We note that the signal waveforms *S*(*t*) have approximately the same shape for all samples, apart from a global scaling factor (see Figure 2 and Figure S1b, Supporting Information). To compare signals from different samples, it is therefore sufficient to consider amplitudes, which are obtained by taking the root mean square (RMS) of the waveform multiplied with the waveform's polarity (±1). We checked that the signal grows linearly with pump power (Figure S2, Supporting Information).

*Evaluating the S2C Strength*: To evaluate the strength of S2C, one needs to extract the amplitude of the charge current.^[^ ^7^ ^]^ We, therefore, consider the relationship between the THz field and the charge‐current density *j*_c_ flowing in the sample plane. In electric‐dipole approximation, the Fourier amplitude of the THz electric field directly behind the sample is given by^[^ ^7^ ^]^

(3) $$\begin{array}{c} E\left(\omega \right)=eZ\left(\omega \right)I_{c}\;\left(\omega \right)=eZ\left(\omega \right)\int dz\;j_{c}\left(z,\omega \right) \end{array}$$

Here, *Z* is the measured sample impedance, which quantifies the charge‐current‐to‐field conversion efficiency. It is found to be approximately constant over the range from 0 to 5 THz (see Figure S3 in the Supporting Information). Because the THz signal is found to grow linearly with the absorbed pump fluence (see Figure S2, Supporting Information), the current density *j*_c_ and, thus, the sheet charge current $I_{c}=\int dz\;j_{c}$ do also.

With these insights, the following procedure is applied to each THz‐signal waveform:^[^ ^29^ ^]^ We i) take the RMS of *S*(*t*) and normalize it by ii) the absorbed pump fluence and iii) the THz impedance *Z*. We, thus, obtain the RMS amplitude of the total sheet charge current *I*_c_ per deposited pump fluence, as shown in **Figures** **3**, **4**, **5** and Figures S4 and S5 (Supporting Information) for various sample parameters and divided by the amplitude of a F|Pt reference sample. Whereas Figure 2 displays typical THz emission signal waveforms, Figures 3, 4, and 5 show normalized THz pulse amplitudes as a function of 3 × 3 different F/N material combinations (Figure 3) and for several interface variations (Figures 4 and 5). The amplitude of the Py|Pt sample in Figures 3, 4, 5 is set to unity. The corresponding values for *Z* and the absorbed pump power can be found in Table S1 (Supporting Information).

**Figure 3:**
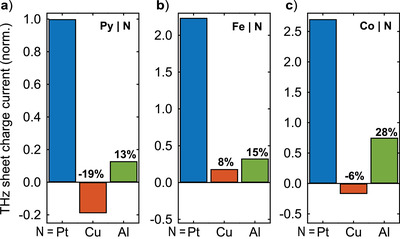
THz charge‐current amplitude from 3  ×  3 different F|N samples. Bars show the root‐mean‐square amplitude of the THz sheet charge current, normalized by the absorbed pump power from a) Py|N, b) Fe|N and c) Co|N bilayers where N is Pt, Cu, or Al. All amplitudes are normalized to the THz emission of Py|Pt. In each panel, the percentage above the F|Cu and F|Al bar quantifies the THz amplitude from these samples relative to that of the respective F|Pt reference bilayer.

**Figure 4:**
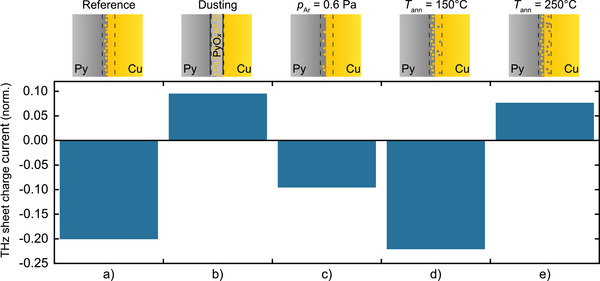
Impact of Py/Cu interface structure on THz emission. Bars show the root‐mean‐square amplitude of the THz sheet charge current, normalized by the absorbed pump power, for Py|Cu bilayers grown under various conditions: a) the Py|Cu reference (see Figure 2), b) Py|PyO*_x_* |Cu with a PyO*_x_* dusting layer (thickness of 0.1 nm), c) Py|Cu deposited under a sputter gas pressure of *p*_Ar_ =   0.6 Pa, and d) Py|Cu ex situ annealed at *T*_ann_ =  150 °C and e) 250 °C. In all configurations, the sample is optically excited from the left‐hand side. The schematics (top row) show the expected qualitative interface structure.

**Figure 5:**
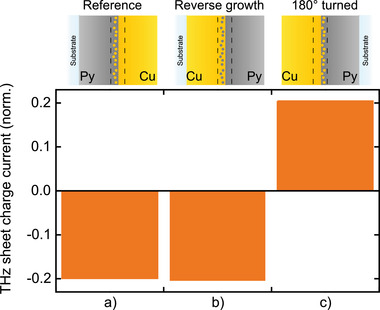
Impact of bilayer growth direction on THz emission. Bars show the root‐mean‐square amplitude of the THz sheet charge current of Py|Cu and Cu|Py bilayers, normalized by the absorbed pump power: a) the Py|Cu reference (see Figure 2), b) Cu|Py grown in reverse order, and c) the physically turned reference sample of panel (a,c). All samples are optically excited from the left‐hand side. The schematics (top row) show the expected qualitative interface structure.

Figure 3 displays the THz‐current amplitude for all combinations of the F‐layer materials Py, Fe, Co and the N‐layer materials Pt, Cu, and Al. In particular, Figure 3a (F = Py) demonstrates that the charge current amplitude of Py|Cu and Py|Al, respectively, amounts to −19% and 13% of that found for Py|Pt. To summarize, for all ferromagnets F, we find sizeable S2C efficiencies on the order of 10% relative to the F|Pt reference sample.

*Impact of F and N Materials*: To discuss the charge‐current amplitudes of Figure 3 in more detail, we make two assumptions. A) Immediately after optical excitation, there is a net spin current from F to N, resulting in a transient decrease of the F magnetization. Therefore, an F/N interface with modified spin transmittance coefficients will only change the magnitude of *j*_s_ at this interface, but not its sign. We consider a violation of this assumption very unlikely. First, if *j*_s_ flowed from N to F, it would increase the magnetic moment of F. Such behavior appears unphysical because the magnetization of the ferromagnets considered here is known to decrease upon heating. Second, previous works on a number of F|N stacks reported a THz peak field whose sign and order of magnitude were consistent with the SHA θ_N_ of the N‐layer material.^[^ ^10^, ^29^ ^]^ This observation indicates that the spin current was always flowing in the same direction, that is, from F to N, directly after optical excitation. B) The total charge current can be written as a sum of S2C in the F‐bulk, N‐bulk, at the F/N interface and at the metal/insulator (I) interfaces (Figure 1b). Using Equations (1) and (3), we, thus, obtain the sheet charge current $I_{c}=\int dz\theta (z)j_{s}(z)$, that is,

(4) $$\begin{array}{c} I_{c}=\left[{\left(\lambda \theta \right)}_{F}+{\left(\lambda \theta \right)}_{N}+{\left(\lambda \theta \right)}_{F/N}\right]\;j_{s0}+I_{c,\text{F/I}} \end{array}$$

where *j*_s0_ is the total spin current density traversing the F/N interface. The λ_*j*_ are effective electron propagation lengths over which S2C takes place (Figure 1a). In the F and N bulk, S2C is due to the ISHE. Prior work suggests that on ultrafast time scales, λ_F_ and λ_N_ can be considered as mean free‐path lengths of electrons^[^ ^30^ ^]^ in F and N, with λ_N_ ≈ 1 nm and 1.9 nm for N = Pt^[^ ^29^ ^]^ and Cu (Figure S9, Supporting Information), respectively, and λ_F_ < 1 nm for F = Fe.^[^ ^8^, ^10^ ^]^ For the F/N interface, the length λ_F/N_ has a less straightforward interpretation. For an ideal interface, λ_F/N_ could be considered as the extension of interface states along *z* or as the mean free path of an electron after it has traversed the interface. For a nonideal interface, one could interpret λ_F/N_ as the thickness of the sheet in which F and N materials are intermixed. Finally, the term *I*_c,F/I_ in Equation (4) accounts for S2C at the F/I interface,^[^ ^22^ ^]^ while S2C at the N/I interface was neglected because the decay length λ_N_ (Figure S9, Supporting Information) is significantly smaller than the N‐layer thickness of 6 nm (see Figure 1a).

We start by considering the ISHE in the N layer. From previous works,^[^ ^9^, ^10^, ^12^, ^13^, ^29^ ^]^ we know that in F|Pt samples, S2C is dominated by the ISHE of Pt. We, therefore, consider the signal from these samples as a reference. For N = Cu and Al, in contrast, the bulk ISHE angle is known to be only a fraction (≈10^−4^…10^−3^) of that of Pt.^[^ ^2^ ^]^ However, in our experiment (Figure 3), we observe one to two orders of magnitude larger signals for N = Cu and Al than what is expected from the strength of the ISHE in the N bulk. We conclude that the signal from F|Cu and F|Al predominantly arises from the ISHE in F and/or from S2C at the F/N and F/I interfaces.

Let us tentatively assume that S2C in F|Cu and F|Al is dominated by the ISHE in F. Due to a possibly different spin transparency of the F/N interface, the magnitude of *j*_s0_ can be different for F|Cu and F|Al. However, the sign of *j*_s0_ remains the same (see assumption (A)), and so does the sign of the charge current *j*_c_ in F. This expectation contradicts the sign change seen for Py|Al versus Py|Cu (Figure 3a) and Co|Al versus Co|Cu (Figure 3c). Furthermore, as both the anomalous Hall and spin Hall angles of Fe and Co are negative,^[^ ^26^, ^31^, ^32^ ^]^ we should obtain the same sign of the THz current in Fe|N and Co|N. This expectation is, again, in contrast to the sign change observed for Fe|Cu and Co|Cu (Figure 3c). Therefore, the data of Figure 3 strongly suggest that the F/N and F/I interfaces make a significant contribution to the S2C in our F|Cu and F|Al bilayer samples.

*Modification of the Py/Cu Interface*: To dedicatedly address the significance of the F/N interface, we varied the interface between Py and Cu by modifying the growth conditions of the Py|Cu stacks as qualitatively indicated by the schematics of Figure 4. First, we dusted the Py/Cu interface by 0.1 nm of Py oxide (PyO*_x_*). When we compare the charge‐current amplitude from the standard Py|Cu bilayer (Figure 4a) to the Py|PyO*_x_* |Cu sample (Figure 4b), we observe a drastic impact: The THz charge current reverses sign, and its magnitude reduces by about 50%. We note that a modified spin transparency of the interface alone would only change the charge‐current magnitude, but not its sign (see assumption (A) in Section 2.4). This result clearly shows that the Py/Cu interface can contribute significantly to S2C in Py|Cu bilayers and can result even in reversal of the sign of the resulting total charge current.

Second, we increased the sputter‐gas pressure from the standard value *p*_Ar_ =  0.3 to 0.6 Pa. The expected effect on the Py|Cu bilayer structure is as follows: In the sputter deposition process, the atoms are emitted from a target due to the impact of argon ions with energies of typically 300 eV.^[^ ^33^ ^]^ On their way to the sample substrate, the energy of the emitted atoms is reduced due to collision cascades, but it remains still far higher than the energy (≈0.2 eV) of thermally evaporated atoms. Upon arrival at the substrate, some of the more energetic atoms are implanted below the surface (see schematic in Figure 4a). This effect is most evident when metals are sputtered on semiconductors or insulators and considered as sputter damage.^[^ ^34^, ^35^, ^36^, ^37^ ^]^ Therefore, when Cu is deposited on top of Py, some of the more energetic Cu atoms are implanted into the Py layer, leading to the asymmetric atomic distribution indicated in Figure 4a. By increasing *p*_Ar_, the Cu atoms are slowed down more strongly by collisions on average before they arrive at the substrate. They are, thus, expected to less likely penetrate into the existing Py layer, resulting in less Cu impurities in the Py interface region (Figure 4c). We find that for the Py|Cu sample grown at 0.6 Pa, the THz signal decreases by about 50% (Figure 4c and Figure S7, Supporting Information), but it maintains its polarity relative to the Py|Cu reference sample (Figure 4a). This result suggests that implantation of less Cu atoms in the Py layer decreases the S2C strength.

Third, following growth, we annealed the Py|Cu reference sample to trigger thermally activated diffusion in the Py/Cu interfacial region.^[^ ^38^, ^39^, ^40^ ^]^ The resulting interface is expected to become more symmetric in terms of the number of Cu defects in the Py layer and Py defects in the Cu layer (see schematics in Figure 4d,e). While an annealing temperature of *T*_ann_ =  150 °C (Figure 4d) results in an increase of the THz emission amplitude of approximately just 10%, annealing at 250 °C has a drastic impact again (Figure 4e and Figure S8, Supporting Information): The THz signal amplitude of the Py|Cu sample changes sign and now agrees with the sign of the Py|Cu sample with oxygen‐dusted interface.

Note that for all the samples considered in Figure 4, the substrate/Py interface is not expected to be modified significantly. We conclude that the massive changes in magnitude and sign of the THz emission amplitude from these samples predominantly arise from S2C at the Py/Cu interface.

*Impact of Growth Direction*: The schematic of Figure 4a suggests that the Py/Cu interface and, potentially, its S2C strength depend on the growth direction of the stack. We, thus, grew Py and Cu in reverse order, and the expected interface structures are qualitatively indicated by the schematics of Figure 5. While for the Py|Cu bilayer, we expect implantation of Cu atoms in Py close to the Py/Cu interface (see Figure 5a), the reverse behavior should occur for Cu|Py (see Figure 5b). This notion is supported by our X‐ray reflectometry measurements which indicate that the interface of the Cu|Py bilayer is substantially smoother than that of the Py|Cu stack (see Section S3 and Figure S6, Supporting Information). The asymmetry of sputtered Py/Cu and Cu/Py interfaces was already observed previously.^[^ ^40^ ^]^ Interface asymmetry is also evident in Pt|Co|Pt and Pd|Co|Pd structures in which the total interface‐induced Dzyaloshinksi–Moriya interaction does not vanish.^[^ ^41^, ^42^ ^]^ Similarly, the exchange anisotropy at the top and bottom interfaces of Py|MnFe|Py has strongly different magnitude.^[^ ^43^ ^]^

Here, we find that the THz emission amplitude from our reversely grown bilayer Cu|Py (Figure 5b) exhibits the same sign and almost the same magnitude as that of the standard Py|Cu sample (Figure 5a). This remarkable behavior is in stark contrast to physically turning the Py|Cu sample by 180° around an axis parallel to the sample magnetization **M** (Figure 5c): The THz signal from the turned sample is a fully reversed version of that from the initial sample (Figure 5a), in agreement with basic symmetry considerations (see Section S1, Supporting Information). We conclude that the Py|Cu bilayer and its counterpart Cu|Py with inverted layer structure are not mirror versions of each other, in agreement with the schematics of the expected qualitative interface structure of the two samples (see Figure 5a,b).

*Suggested Scenario of Interfacial S2C*: We ascribe the observations of Figures 4 and 5 to skew scattering^[^ ^2^, ^44^ ^]^ of laser‐excited spin‐polarized electrons off structural imperfections at the F/N interface (**Figure** **6**a). These scattering centers exhibit a considerably different spin‐orbit coupling relative to their environment. In our samples, they can, for instance, arise from oxygen interface dusting (Figure 4b), from Cu impurities in Py (abbreviated Py(Cu)) and from Py impurities in Cu (short Py(Cu)) (Figure 5a,c). Note that the difference of the number of valence electrons and, thus, of the spin‐orbit coupling of host and impurity material in Py(Cu) versus Cu(Py) have opposite sign.^[^ ^45^ ^]^ We, therefore, expect that skew scattering angles and the strength θ of S2C have opposite sign as well (Figure 6b).

**Figure 6:**
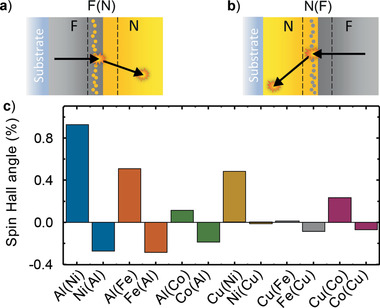
Possible S2C by skew scattering at interfacial imperfections. a) Growth of N = Cu on F = Py leads to an interfacial layer Py(Cu) of Cu atoms in a Py matrix. This layer gives rise to skew scattering of the laser‐excited spin‐polarized electrons originating from the Py layer. The black arrows indicate the mean velocity of an electron before and after traversal of the interface. The orange symbols represent scattering events. Note that the transverse charge current is enhanced by a long electron mean free path in N. b) Same as (a) but with roles of Cu and Py exchanged. Note that the bilayers of panels (a,b) are not mirror versions of each other, and the Py(Cu) and Cu(Py) interface layers are expected to exhibit spin Hall angles of opposite sign. c) Calculated spin‐Hall angle for 1 atom% of A impurities in a host material B, denoted as B(A). In the convention used here, the spin‐Hall angle of Pt is positive and of the order of 10%.

Our model along with the expected distribution of Py(Cu) and Cu(Py) impurities in the various samples can consistently explain all our observations of Figures 4 and 5: When the number of Py(Cu) impurities decreases due to a higher sputter‐gas pressure, the strength of S2C does also (Figure 4c). Likewise, when Cu(Py) impurities are added by annealing, they compensate and eventually exceed the S2C due to the Py(Cu) impurities, ultimately thereby resulting in a polarity change of the THz emission signal (Figure 4d,e). Finally, in the Py|Cu and Cu|Py samples grown in reverse order, Py(Cu) and Cu(Py) impurities are expected to prevail, respectively (Figure 6a,b). Therefore, values of θ with opposite sign result. Because the spin current has opposite direction, Py|Cu and Cu|Py samples deliver THz emission amplitudes of the same polarity (Figure 5a,b).

*Model Calculations*: To put the scenario of Figure 6a,b on a more quantitative basis, we calculated the SHA of F(N) and N(F) alloys considering skew scattering as the only S2C process. We assumed a plausible impurity‐atom fraction of 1%, which coincides with the dilute limit for which scattering from different impurities can be considered independent. The results of the calculations are displayed in Figure 6c.

First, the sign of the calculated SHA of F(N) and N(F) is always opposite, in agreement with the qualitative arguments in Section 2.7 and with our experimental observations for reversely grown samples (see Figure 5 and Figures S7 and S8, Supporting Information).

Second, Cu(Py) defects cause significantly stronger and opposite deflection than Py(Cu) defects (Figure 6c). This behavior can well explain the sign change of the overall S2C of the as‐grown Py|Cu sample upon annealing (see Figure 5a,d,e).

Third, to compare the order of magnitude of measured and calculated S2C, we estimate the SHA from our measurements. We assume S2C in Py|Pt is dominated by the Pt bulk (*I*_c_(PyPt) = *j*_s0_λ_Pt_θ_Pt_), whereas in Py|Cu, it is dominated by the Py bulk and the Py/Cu interface (*I*_c_(PyCu) = *j*_s0_λ_Py_θ_Py_ + *j*_s0_λ_Py/Cu_θ_Py/Cu_, see Section 2.5). We obtain

(5) $$\begin{array}{c} \theta_{\text{Py/Cu}}\;\;\approx \;\;\frac{I_{c}\left(\text{PyCu}\right)-I_{c}\left(\text{CuPy}\right)}{I_{c}\left(\text{PyPt}\right)}\cdot \theta_{\text{Pt}}\cdot \frac{\lambda_{\text{Pt}}}{\lambda_{\text{Py}/\text{Cu}}} \end{array}$$

The difference *I*_c_(PyCu) − *I*_c_(CuPy) cancels the contribution of S2C in the Py layer (*j*_s0_λ_Py_θ_Py_), which is expected to be the same for the two samples. In Equation (5), the first factor is ≈0.2 (Figure 3a), θ_Pt_ ≈ 0.1^[^ ^2^, ^46^, ^47^ ^]^ and λ_Pt_ =  1 nm,^[^ ^10^, ^29^ ^]^ and the effective extension λ_Py/Cu_ of the interface region is taken to be on the order of 1 nm.^[^ ^48^, ^49^ ^]^ We obtain θ_Py/Cu_ =  2%, which is in good agreement with the order of magnitude of the calculated SHA of Ni(Al), Al(Ni), Fe(Al), and Al(Fe). A similar conclusion can be drawn for the other systems shown in Figure 3.

Note that Equation (1) implies a local S2C scenario, that is, *j*_c_ is determined by *j*_s_ at the very same position *z*. While this approach is appropriate for the intrinsic ISHE mechanism, the skew‐scattering scenario of Figure 6a is actually nonlocal: The charge current *j*_c_ behind the interface is determined by the wavevector change due to skew scattering right at the imperfect Py/Cu interface. Therefore, the transverse motion of the electron persists until the next scattering event in the “cleaner” bulk of the Cu layer occurs. In this picture, the characteristic length λ_Py/Cu_ is rather given by the mean free path of the electron in Cu, which equals 1.9 nm (see Figure S9. Supporting Information). With this refined consideration, Equation (5) yields θ_Py/Cu_ =  1%, which agrees even better with the calculated values of the SHA. This value may still be overestimated because our analysis neglects a possible spin memory loss at the Py/Cu interface^[^ ^50^ ^]^ (Equation (4)) and the contribution *I*_c,Py/I_ (Equation (5)). We conclude that the order of magnitude of the measured S2C strength θ_F/N_ of the F/N interfaces (Figure 3) is in good agreement with the calculated values of the SHA of N(F) and F(N) materials (Figure 6d).

*Discussion*: Our model calculations are consistent with the observations of Figures 4 and 5 and the order of magnitude of the F–N interfacial S2C contribution. We did, however, not attempt to compare the signs of measured THz charge currents and calculated SHAs for all samples for two reasons. First, the actual interface structure (F(N) vs N(F)) is not known and may vary when the F or N material is changed. Second, the ISHE of the F layer may make another contribution to S2C and so add an offset to the measured THz charge current. The same argument applies to a contribution to the THz charge current from ultrafast demagnetization.^[^ ^51^ ^]^

Regarding other S2C mechanisms, we cannot fully exclude contributions from the side‐jump scenario^[^ ^52^ ^]^ or the IREE.^[^ ^5^, ^6^ ^]^ However, a sizeable IREE appears to be rather unlikely. First, the IREE requires strongly Rashba‐split interface states that are not trivially available in our samples since the N‐layer materials Cu and Al lack strong spin‐orbit interaction.^[^ ^53^ ^]^ Second, from the Rashba perspective, the two Cu|Py samples of Figure 5b,c are approximately identical. Thus, a sign change of the IREE in these two samples is rather unexpected, in contrast to the experimental observation. Third, the good agreement of our experimental data with the calculated skew‐scattering contribution strongly suggests that the other sources of S2C play a minor role in our samples.

In conclusion, we observed sizeable S2C induced by interfaces of F|N bilayers with weak bulk spin‐orbit coupling, as large as 20% of S2C in F|Pt reference layers. Our results have important implications. First, they show that interfacial contributions to S2C need to be considered before the measured magnetization‐dependent transverse charge current is assigned exclusively to bulk effects in the F or N layer. Second, interfacial S2C can arise from effects beyond the usually considered IREE mechanism. Sign and order of magnitude of the interfacial S2C observed here are consistent with a dominant role of skew scattering of spin‐polarized electrons at F(N) and/or N(F) interface layers (Figure 6a). Third, the skew scattering off Cu(Py) interfacial imperfections (Figure 6b) is enhanced by the relatively long relaxation length (λ_Py/Cu_ ≈ 1.9 nm) of the ballistically propagating electrons in the Cu layer (Figure S9, Supporting Information). This remarkable nonlocal mechanism opens up a promising route to enhancing S2C by controlling the structure of the spintronic interface.

## Experimental Section

### Sample Preparation

The thin‐film stacks were deposited using a fully automated Singulus Rotaris deposition system equipped with 12 magnetron targets (100 mm diameter) and a 200 mm wafer handler that permits highly reproducible and uniform deposition. The glass substrates were glued with Kapton tape on a wafer carrier, leaving about 1/3 of the substrate area undeposited for reference measurements. Deposition rates of the individual materials were calibrated using X‐ray reflectometry, and the respective sputtering times were adjusted to achieve the nominal layer thicknesses. Metals were DC‐sputtered whereas the MgO cover layer was RF‐sputtered. The sputtering gas was pure argon except for the PyO*_x_* dusting layer, for which nominally 0.1 nm thick Py layer was deposited in a mixed Ar:O_2_ flow with a volume ratio of 4:1. It is expected that the oxygen partial pressure in the gas atmosphere leads not only to an oxidation of all the transition metal ions deposited in this reactive atmosphere, but also of the topmost existing surface layers such that at least two monolayers at the interface are oxidized.

### Interface Tailoring

To achieve different interface qualities, films at different sputter gas pressures and different ex situ annealing were deposited. Changing the argon gas flow changes the background pressure from 0.3 to 0.6 Pa. Thus, the collision probability of sputtered atoms with the background gas is enhanced by a factor of 2, leading to a smaller number of high‐energy atoms arriving at the substrate and accordingly a lower degree of implantation.

Thermally driven rearrangement of atoms is a diffusion process and should lead to a symmetric interface because the transition metal atoms Cu, Ni, and Fe are of similar size. Thermal annealing was implemented by heating of some of the samples in a vacuum annealing oven at a pressure of less than 10^−4^ Pa to temperatures of 150 °C and 250 °C, respectively. The temperature was ramped up to the final temperature within 1 h and maintained for 2 h before the heater was switched off.

Note that variation of the sputter power would have a relatively little impact on the sample structure because 800 W (499 V  ×  1.6 A) and 200 W (408 V  ×  0.49 A) of power result in a similar energy of the sputtered particles while the deposition time scales inversely with the current.

### THz‐Emission Setup

The in‐plane sample magnetization **M** was saturated by an external magnetic field of 30 mT. As schematically shown in Figure 1a, the sample was excited by linearly polarized laser pulses (energy 1 nJ, duration 10 fs, center wavelength 800 nm, repetition rate 80 MHz) from a Ti:sapphire laser oscillator under normal incidence from the glass side. The beam diameter at the sample was 22 µm full width at half‐maximum of the intensity, as determined by a pinhole method.

The THz electric field emitted in transmission direction was detected by electro‐optic sampling,^[^ ^25^ ^]^ where probe pulses (0.6 nJ, 10 fs) from the same laser co‐propagate with the THz field through an electro‐optic crystal. The resulting signal *S*(*t*) equals twice the THz‐field‐induced probe ellipticity, where *t* is the delay between the THz and sampling pulse. As electro‐optic sensor, a ZnTe(110) crystal with a thickness of 1 mm was used. If not mentioned otherwise, all measurements were performed at room temperature under ambient conditions.

### Sample Characterization

Structural characterization of the sample was done by X‐ray reflectivity measurements (see Section S3, Supporting Information). The samples were also characterized in terms of optical absorptance and THz and/or DC electrical transport measurements. By measuring the fractions *R* and *T* of, respectively, reflected and transmitted power of the pump beam, the sample absorptance *A* was determined by *A*  =  1 − *R* − *T*. It is listed for all samples in Table S1 (Supporting Information). From the spot diameter at the sample position (see above) and the laser repetition rate, the pump fluence is found that is absorbed by the F|N stack.

For a subset of samples, the electrical impedance *Z* of the metal stack was determined by THz transmission spectroscopy. THz pulses were generated by exciting a spintronic THz emitter^[^ ^10^ ^]^ with optical pulses from the same laser as in the THz‐emission experiments and by focusing the THz pulses on the sample under investigation. The field of the THz pulses having traversed the sample was characterized by electro‐optic detection in a GaP(110) crystal (thickness of 250 µm). By conducting a reference measurement on substrate regions without metal film, the impedance of the metal stack is determined (see Section S2, Supporting Information). The residual pump beam from the THz pulse generation was blocked by a Si wafer.

Alternatively, the sample impedance *Z* was determined by measuring the DC sheet resistance *R*_DC_ of the sample film by a van‐der‐Pauw‐type approach. From *R*_DC_ and the refractive index of the substrate material, the value of *Z* was inferred.

### SHA Calculations

All transport calculations are based on the solution of a linearized Boltzmann equation including vertex corrections and assuming the limit of diluted impurity concentrations.^[^ ^45^ ^]^ The input parameters were calculated from a fully relativistic Korringa–Kohn–Rostoker Green's‐function method within density‐functional theory and exploiting the local density approximation.^[^ ^54^ ^]^ The impurity problem was solved on a real space cluster with a central substitutional impurity embedded in the infinite and perfect host crystal.^[^ ^55^ ^]^

## Conflict of Interest

The authors declare no conflict of interest.

## Supporting information

Supporting Information

## Data Availability

The data that support the findings of this study are available from the corresponding author upon reasonable request.
