# Supplementary material for: Terahertz Spin‐to‐Charge Conversion by Interfacial Skew Scattering in Metallic Bilayers
Source: Adv Mater. 2021 Jan 27;33(9):2006281. doi: 10.1002/adma.202006281 (PMC11469024; doi:10.1002/adma.202006281)
Supplement: Supplementary file 1 — Supporting Information [file ADMA-33-2006281-s001.pdf]

© 2021 Wiley-VCH GmbH

ADVANCED MATERIALS

Supporting Information

for *Adv. Mater.*, DOI: 10.1002/adma.202006281

Terahertz Spin-to-Charge Conversion by Interfacial Skew Scattering in Metallic Bilayers

Oliver Gueckstock, Lukáš Nádvorník,* Martin Gradhand, Tom Sebastian Seifert, Genaro Bierhance, Reza Rouzegar, Martin Wolf, Mehran Vafaee, Joel Cramer, Maria Andromachi Syskaki, Georg Woltersdorf, Ingrid Mertig, Gerhard Jakob, Mathias Kläui, and Tobias Kampfrath*

Supporting information

Terahertz spin-to-charge conversion by interfacial skew scattering in metallic bilayers

Oliver Gueckstock, Lukáš Nádvorník,* Martin Gradhand, Tom Sebastian Seifert, Genaro Bierhance, Reza Rouzegar, Martin Wolf, Mehran Vafaee, Joel Cramer, Maria Andromachi Syskaki, Georg Woltersdorf, Ingrid Mertig, Gerhard Jakob, Mathias Kläui, Tobias Kampfrath*

S1 Basic symmetry considerations

We analyze what happens to the THz emission signal when the F|N sample is turned by 180° about an axis parallel to the F-layer magnetization $\boldsymbol{M}$. According to our experimental geometry (Figure 1a), the THz emission originates from an in-plane charge current $\boldsymbol{I}_\mathrm{c} = \int \mathrm{d}z\, \boldsymbol{j}_\mathrm{c}(z)$ that is quadratic in the pump electric field $\boldsymbol{E}_\mathrm{p}$ (Figure S2b). Because $\boldsymbol{E}_\mathrm{p}$ is approximately constant over the thickness of the F|N stack (Figure 1a), we can write

$$I_{\mathrm{c}i} = \sum_{j,k=1}^{3} d_{ijk}(\boldsymbol{M}) E_{\mathrm{p}j} E_{\mathrm{p}k}. \qquad (1)$$

where the response function $d_{ijk}(\boldsymbol{M})$ is a 3rd-rank tensor depending on $\boldsymbol{M}$. The indices i, j and k can take the values $1, 2, 3$, which refer to the x, y and z Cartesian components, respectively. In Eq. (6), the time dependence of $I_{\mathrm{c}i}$, d_{ijk}, $E_{\mathrm{p}j}$ and $E_{\mathrm{p}k}$ and the integration over the time arguments of $E_{\mathrm{p}j}$ and $E_{\mathrm{p}k}$ is suppressed because they are not relevant for what follows.

We choose the z-axis parallel to the surface normal of the sample, and the magnetization is chosen to point along the x-axis. Therefore, a rotation by 180° about an axis parallel to $\boldsymbol{M}$ is described by the diagonal matrix $\underline{R}^x = \mathrm{diag}(1, -1, -1)$. Upon rotation of the sample, the response function of the sample transforms like a polar tensor according to[S1]

$$d'_{i'j'k'}(\boldsymbol{M}') = \sum_{i,j,k=1}^{3} R^x_{i'i} R^x_{j'j} R^x_{k'k} d_{ijk}(\boldsymbol{M}). \quad (2)$$

Because of $\boldsymbol{M}' = \underline{R}^x \boldsymbol{M} = \boldsymbol{M}$ and because $\underline{R}^x$ is diagonal, the summation in Eq. (7) is restricted to $i' = i$, $j' = j$ and $k' = k$, resulting in the simple product

$$d'_{ijk}(\boldsymbol{M}) = R^x_{ii} R^x_{jj} R^x_{kk} d_{ijk}(\boldsymbol{M}). \quad (3)$$

In our experiment, $\boldsymbol{I}_{\mathrm{c}}$ is observed to be directed along the y-axis (perpendicular to $\boldsymbol{M}$), and $\boldsymbol{E}_{\mathrm{p}}$ is oriented along either the x- or y-axis. We are, thus, only interested in d'_{ykk} with $k = 1$ or 2, for which Eq. (8) becomes

$$d'_{ykk}(\boldsymbol{M}) = -d_{ykk}(\boldsymbol{M}). \quad (4)$$

We conclude that under the conditions of our experiment, the thickness-integrated charge current reverses sign when the sample is turned by 180° about an axis parallel to the sample magnetization.

S2 Samples: transport characterization

We characterized our samples in terms of optical absorptance as well as electrical transport at DC and THz frequencies. The results are summarized in Table S1. The absorptance A quantifies how well the incident pump pulse is coupled into the metal film.

Electrical transport of all samples is characterized either by contact-free THz transmission measurements (yielding broadband conductance) or by the van-der-Pauw method (yielding the DC conductance).[S2]

In brief, THz transmission measurements primarily yield the THz transmission [S3]

$$T(\omega) = 2n_1(\omega)\frac{Z(\omega)}{Z_0} = 2n_1(\omega)\frac{1}{n_1(\omega) + n_2(\omega) + Z_0 G(\omega)} \quad (5)$$

from which the frequency-dependent impedance $Z(\omega)$ of the whole sample (metal plus substrate) and the sheet conductance $G(\omega)$ of the metal stack can be extracted. In Eq. (10), n_1 and $n_2 \approx 1$ denotes, respectively, the refractive index of the substrate material and air, and $Z_0 \approx 377\ \Omega$ is the free-space impedance. The sheet conductance

$$G(\omega) = \int_0^d \mathrm{dz}\, \sigma(\omega, z) = \bar{\sigma}(\omega) d \quad (6)$$

of the metal stack equals the metal conductivity $\sigma(\omega, z)$ integrated over the stack thickness d. For the samples studied here, $T(\omega)$ and $G(\omega)$ vary relatively little over the measured frequency window from 1 THz to 5 THz (see Figure S3). The mean DC and THz sheet impedance are summarized in Table S1.

We assume for the metal stack that the mean conductivity of the metal stack as defined by Eq. (11) can be adequately described by the Drude formula

$$\bar{\sigma}(\omega) = \frac{\sigma_0}{1 - \mathrm{i}\omega\tau} \quad (7)$$

where σ_0 is the mean DC conductance and τ is the mean velocity relaxation time. We use Eqs. (10), (11) and (12) to fit the measured THz transmission $T(\omega)$ with σ_0 and τ as fit parameters.

S3 Samples: structure characterization

Asymmetric growth Cu|Py vs Py|Cu: During sputter deposition of multilayers, the order of F and N deposition can crucially influence the resulting roughness and interface quality, depending on the wetting behavior of the materials with each other and the substrate and on the bombardment

with energetic particles. In each sputtering run, we prepared samples on fused silica substrates for the THz emission experiments and control samples on thermally oxidized Si wafers. Thus, in both cases the metal layers are deposited on amorphous SiO_2.

The control samples were investigated by X-ray reflectometry. The nonzero thicknesses of the layers lead to an oscillatory X-ray intensity as a function of the incident angle Θ of the x-ray beam with respect to the sample surface that is reflected at an angle 2Θ with respect to the incoming beam (Figure S6). The number of visible interference fringes is a direct measure of the interface quality of the layer structure.

In Figure S6a, we compare a Cu|Py sample with nominal layer sequence Si||SiO_2(100 nm)|Cu(6 nm)|Py(3 nm)|MgO(3 nm) to a Py|Cu sample with nominal layer sequence Si||SiO_2 (100 nm)|Py(3 nm)|Cu(6 nm)|MgO(3 nm). By qualitative inspection, the Cu|Py sample (blue line) shows much clearer interference fringes than the Py|Cu sample (red line), thus indicating a significantly smoother interface with less interlayer diffusion.

To achieve more quantitative insights, the reflectometry data were modelled using GenX software[S4]. Significant differences in the roughness parameters were needed to reproduce the experimental data. The smallest least-square errors were achieved for the sample-dependent parameters shown in Table S2.

Note that the extracted roughness of the Py/Cu interface of Py|Cu is 5 to 6 times higher than that of the Cu/Py interface of Cu|Py, thus demonstrating the absence of symmetry while reversing the layer order. These numbers certainly need to be taken with some caution because fitting of the reflectometry data is model-dependent. For instance, the deposition times of the respective materials were equal for both samples and, thus, the total number of deposited atoms should be the same. This expectation is not fully reproduced by the simulations. Likewise, the large roughness of the Py layer does not allow for a Gaussian thickness fluctuation as negative

thickness values are unphysical. The simulation rather assumes roughness considerably smaller than the respective layer thickness.

Ex-situ annealing of Cu|Py vs Py|Cu: In Figure S6b, we compare X-ray reflectivity data from the as-deposited Py|Cu bilayer and after annealing at 250 °C. As with Figure S6a, we set some constraints for the simulation which are summarized in Table S3. Interestingly, the interface does not become smoother by annealing. Because the X-rays probe thickness fluctuations on the length scale of their coherence length (~1 μm), the fitted roughness is not necessarily related to the implantation of single atoms at the boundary. Therefore, these results do not contradict our model of Figure 6c.

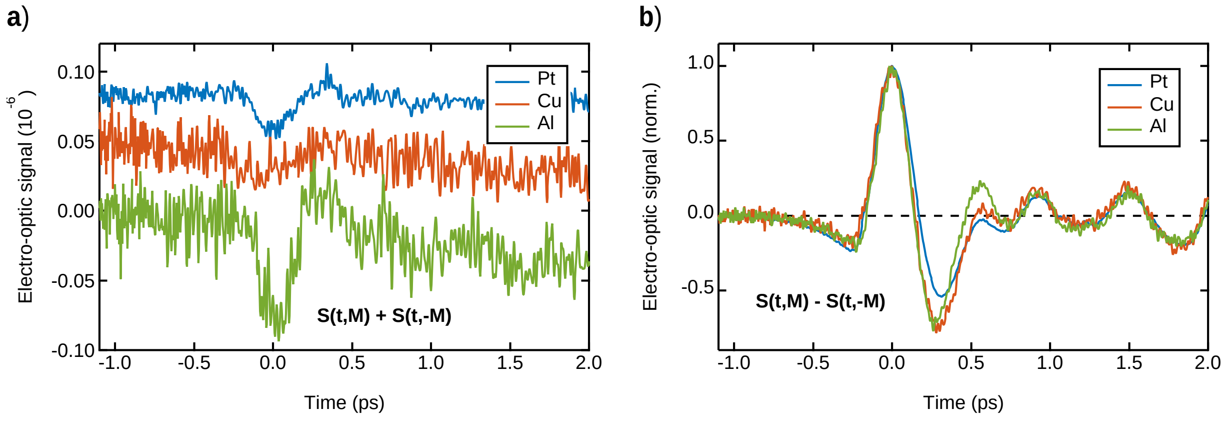

Figure S1. THz-emission raw data from Py|N. a) Signal component $S(t,\boldsymbol{M})+S(t,-\boldsymbol{M})$ even in the external magnetic field for F=Pt, Al and Cu. It is two orders of magnitude smaller than the odd component shown in Fig. 2 of the main text. b) Normalized signal component $S(t)=S(t,\boldsymbol{M})-S(t,-\boldsymbol{M})$ odd in the external magnetic field for the same N materials as in panel a). The temporal dynamics of the N materials shown are approximately the same.

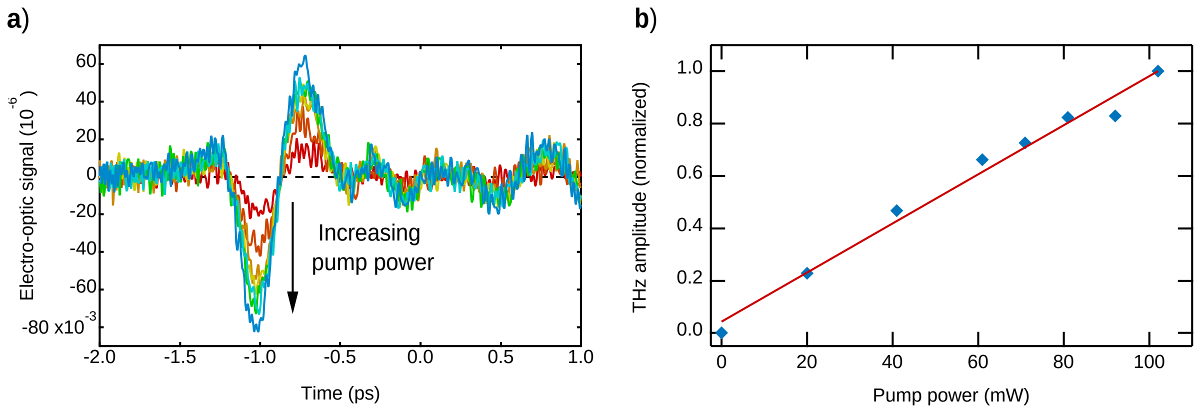

Figure S2. Pump-power dependence of THz emission. a) Time-domain electro-optic THz emission signals from Py|Cu. The signal amplitude increases with increasing pump power. b) Dots show the root-mean-square of the waveforms from panel a). The data are fitted with a linear function as depicted by the red line.

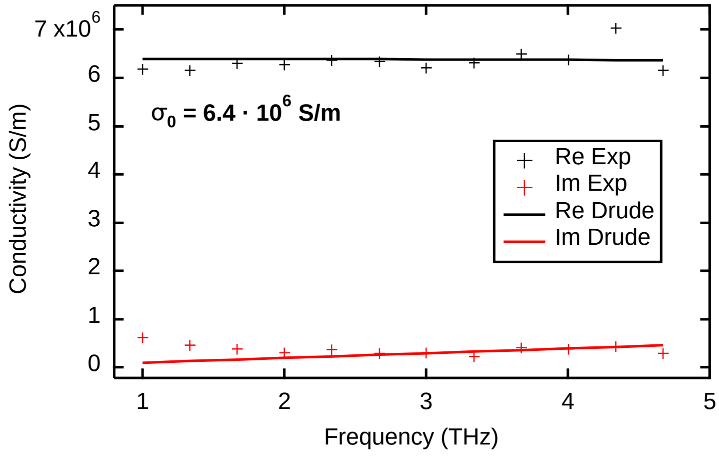

Figure S3. Conductivity of Py|Cu. Real and imaginary part of the measured conductivity vs frequency along with a Drude fit (solid lines). The conductivity is approximately constant over the measured frequency range of 0 to 5 THz. In addition, $\boldsymbol{\sigma_0}$ is the measured DC conductivity of the sample.

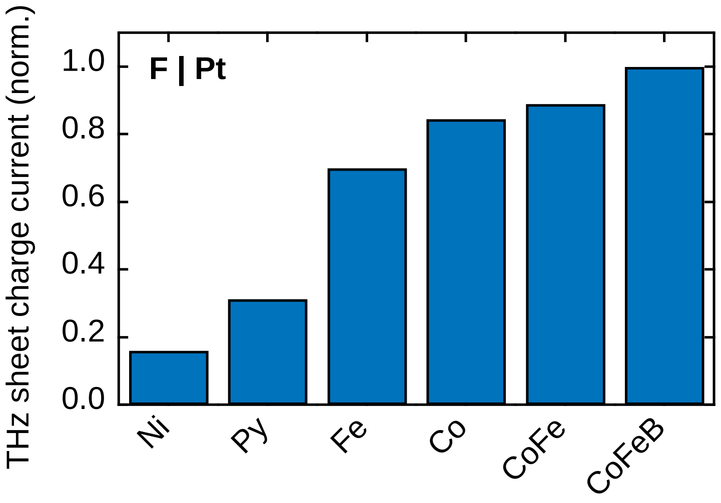

Figure S4. THz charge-current amplitudes vs F. Bars show the root-mean square of the THz sheet charge current of various F|Pt samples with F=Ni, Py, Fe, Co, $Co_{70}Fe_{30}$ and $Co_{20}Fe_{60}B_{20}$, normalized by the absorbed pump power. These data are in good agreement with previous measurements[S3].

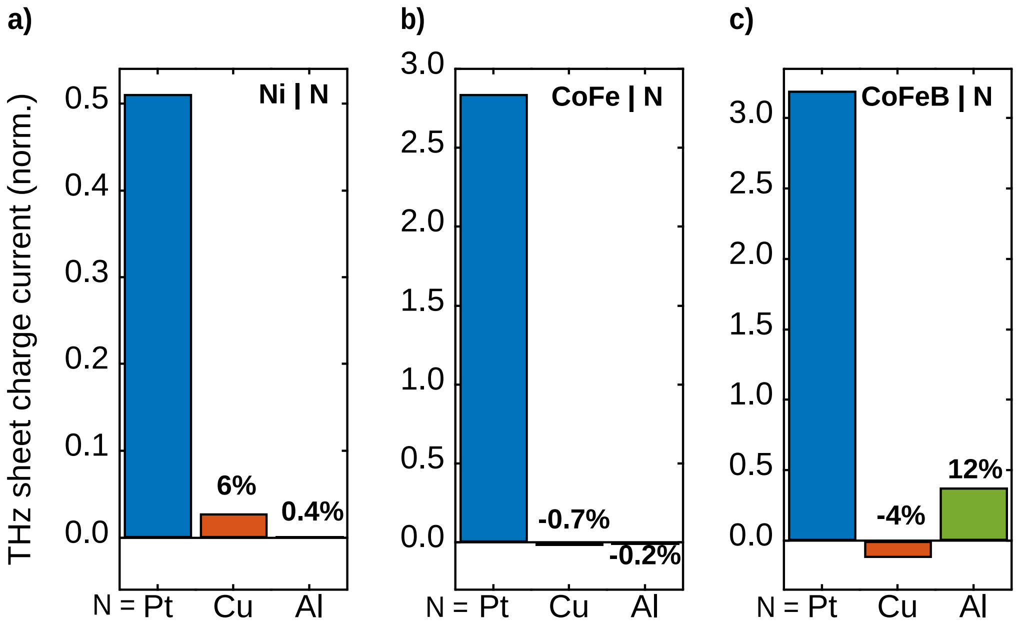

Figure S5. THz charge-current amplitude from 3 × 3 more F|N samples. Bars show the root-mean square of the THz sheet charge current, normalized by the absorbed pump power from a) Ni|N, b) $Co_{70}Fe_{30}$|N and c) $Co_{20}Fe_{60}B_{20}$|N bilayers where N=Pt, Cu or Al. All curves are normalized with respect to the THz emission of Py|Pt. In each panel, the percentages refer to the amplitude of the respective F|Pt.

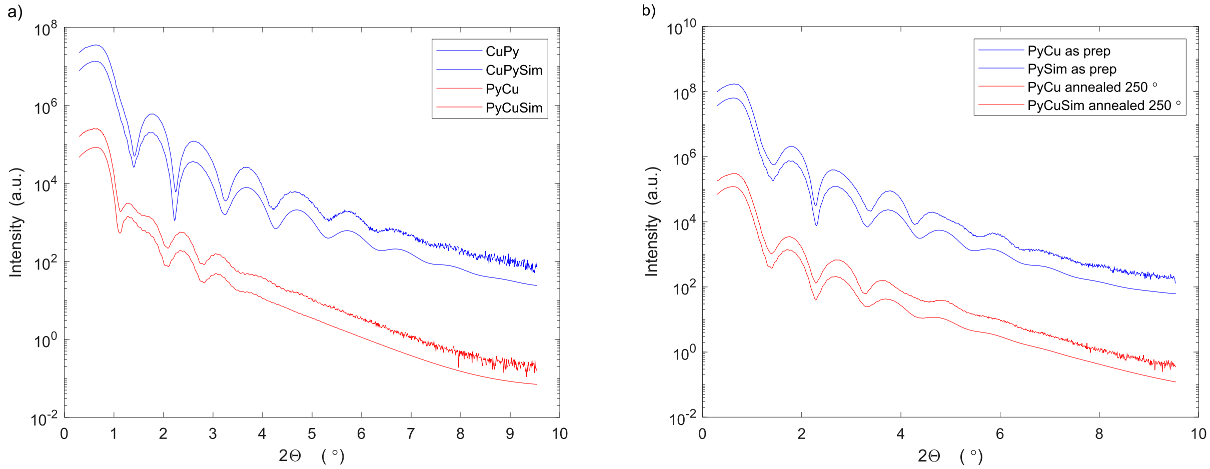

Figure S6. X-ray reflectivity of Py|Cu and Cu|Py. Measured and simulated X-ray reflectance vs reflection angle 2Θ for a) as deposited Cu|Py (blue lines) and Py|Cu samples (red lines) on thermally oxidized Si substrates. b) Py|Cu sample as deposited and after annealing at 250 °C. The measured data are shifted by a factor of 100, and the respective simulations below the experimental curves are shifted by a factor of 3 for clarity.

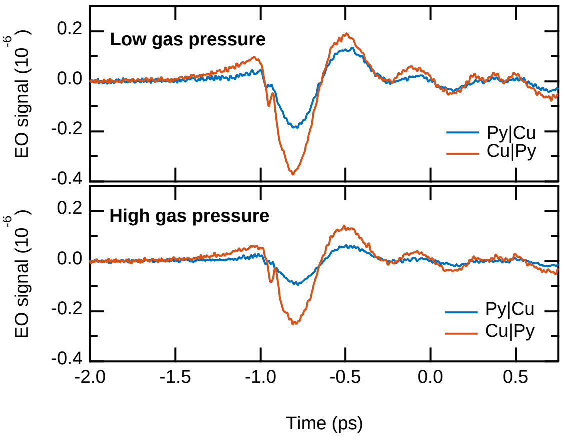

Figure S7. THz emission waveforms for Cu|Py and Py|Cu samples grown at low (0.3 Pa) and high (0.6 Pa) sputter gas pressure.

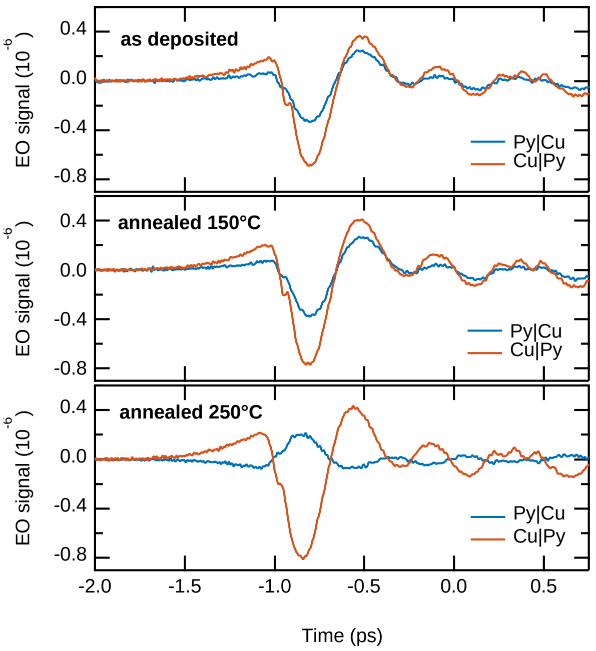

Figure S8. THz emission waveforms for Cu|Py and Py|Cu samples as grown and ex-situ annealing at 150 °C and 250 °C.

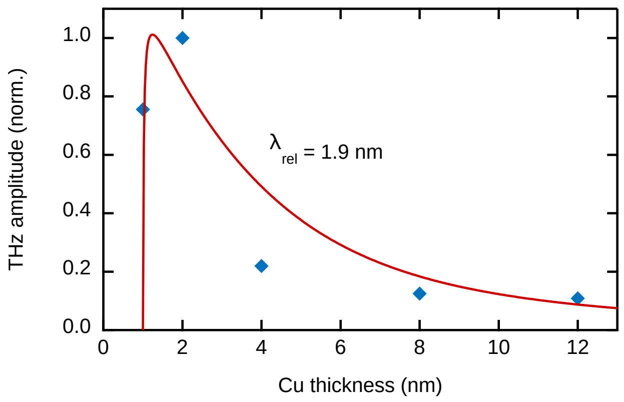

Figure S9. THz emission amplitude (root mean square) from Py|Cu samples as function of the Cu-layer thickness. The red solid line depicts a fit based on the equations in Seifert *et al.*, J. Phys. D: Appl. Phys.51 364003 (2018)[S5]. We obtain a relaxation length of $\lambda_{\mathrm{rel}} = 1.9$ nm.

Sample	Absorptance A	THz sheet impedance Z (Ω)	Mean conductivity σ_0 (MS/m)	Mean Drude scattering time τ (fs)
**Py|Pt**	62 %	34	2.5	58
**Py|Cu**	49 %	15	6.5	11
**Py|Al**	62 %	32	2.7	/
**Fe|Pt**	66 %	17	5.8	18
**Fe|Cu**	64 %	57	1.2	33
**Fe|Al**	65 %	16	6.3	15
**Co|Pt**	63 %	26	3.6	53
**Co|Cu**	57 %	11	9.4	12
**Co|Al**	62 %	24	3.9	83
**Py|Cu**	47 %	16	6.4	15
**Py|PyOx|Cu**	47 %	17	5.6	71
**Cu|Py**	56 %	31	2.9	50
**Sample**	**Absorptance A**	**THz sheet impedance Z (Ω)**	**Mean DC conductivity σ_{DC} (MS/m)**	
**Ni|Pt**	66 %	31	2.9	
**Ni|Cu**	53 %	35	2.4	
**Ni|Al**	62 %	64	1.0	
**CoFe|Pt**	65 %	39	2.1	
**CoFe|Cu**	39 %	34	2.5	
**CoFe|Al**	60 %	37	2.3	
**CoFeB|Pt**	69 %	33	2.6	
**CoFeB|Cu**	53 %	12	8.3	
**CoFeB|Al**	64 %	28	3.2	
**Py|Cu (as. dep.)**	47 %	16	6.3	
**Cu|Py (as. dep.)**	54 %	31	2.8	
**Py|Cu (150 °C)**	49 %	15	6.5	
**Cu|Py (150 °C)**	54 %	34	2.4	
**Py|Cu (250 °C)**	50 %	20	4.6	
**Cu|Py (250 °C)**	54 %	33	2.4	

Table S1. Sample parameters. Absorbed pump power, sheet impedance, mean conductivity and mean Drude scattering time for all samples studied within this work. The error of the optical absorptance measurements is on the order of 2% for all samples. The conductivity values are obtained either by THz transmission measurements σ_0 (see Figure S3) or by the van der Pauw method (σ_{DC}) using Eqs. (10) and (11). The Drude scattering times are obtained from fits.

Parameter	Cu|Py	Py|Cu
MgO thickness (nm)	4.2	3.1
MgO roughness (nm)	1.0	1.0
MgO density (g/cm^3)	3.1	3.2
Py thickness (nm)	2.0	4.2
Py roughness (nm)	0.5	3.2
Py density (g/cm^3)	8.5	8.6
Cu thickness (nm)	6.4	5.7
Cu roughness (nm)	0.6	1.0
Cu density (g/cm^3)	9.0	8.2
SiO_2 thickness (nm)	97	96
SiO_2 roughness (nm)	0.3	0.4
Si roughness (nm)	0.8	0.7

Table S2. Sample parameters from the X-ray reflectometry simulation for Cu|Py vs Py|Cu. The smallest least-square errors were achieved for the shown sample-dependent parameters using GenX software[S4].

Parameter	Py|Cu	Py|Cu (250 °C)	Constraints
MgO thickness (nm)	4.0	3.8	2.2…4.0
MgO roughness (nm)	1.2	1.5	
MgO density (g/cm^3)	3.5	3.4	3.0…3.5
Py thickness (nm)	2.5	2.7	2.5…3.5
Py roughness (nm)	3.9	4.0	< 4.0
Py density (g/cm^3)	8.8	8.8	8.3…8.8
Cu thickness (nm)	5.7	5.6	5.5…6.5
Cu roughness (nm)	0.6	0.7	
Cu density (g/cm^3)	8.7	8.5	8.5…9
SiO_2 thickness (nm)	95	97	
SiO_2 roughness (nm)	0.3	0.3	
Si roughness (nm)	0.8	0.8	

Table S3. Sample parameters from the X-ray reflectometry model for Py|Cu as deposited and annealed at 250 °C. We set the shown constraints in the GenX software[S4] to obtain the smallest least-square errors for each parameter.

References

[S1] R. R. Birss, *Symmetry and Magnetism*, North-Holland Publishing Company, 1966.

[S2] L. J. van der Pauw, Philips Technical Review 1958, 20.

[S3] T. Seifert, S. Jaiswal, U. Martens, J. Hannegan, L. Braun, P. Maldonado, F. Freimuth, A. Kronenberg, J. Henrizi, I. Radu, E. Beaurepaire, Y. Mokrousov, P. M. Oppeneer, M. Jourdan, G. Jakob, D. Turchinovich, L. M. Hayden, M. Wolf, M. Münzenberg, M. Kläui, T. Kampfrath, Nature Photonics 2016, 10, 483.

[S4] M. Björck, G. Andersson, Journal of Applied Crystallography 2007, 40, 1174.

[S5] T. S. Seifert, N. M. Tran, O. Gueckstock, S. M. Rouzegar, L. Nadvornik, S. Jaiswal, G. Jakob, V. V. Temnov, M. Münzenberg, M. Wolf, M. Kläui, T. Kampfrath, Journal of Physics D: Applied Physics 2018, 51, 364003.
